# Urine Metabolomic Profile of Breast- versus Formula-Fed Neonates Using a Synbiotic-Enriched Formula

**DOI:** 10.3390/ijms231810476

**Published:** 2022-09-09

**Authors:** Vasiliki Falaina, Charalambos Fotakis, Theodora Boutsikou, Thalia Tsiaka, Georgios Moros, Sotirios Ouzounis, Vasiliki Andreou, Zoi Iliodromiti, Theodoros Xanthos, Yvan Vandenplas, Nicoletta Iacovidou, Panagiotis Zoumpoulakis

**Affiliations:** 1 Department of Neonatology, Medical School, National and Kapodistrian University of Athens, 157 72 Athens, Greece; 2 Neonatal Intensive Care Unit, General Hospital of Nikaia, Piraeus “Agios Panteleimon”, 184 54 Piraeus, Greece; 3 Institute of Chemical Biology, National Hellenic Research Foundation, 116 35 Athens, Greece; 4 Department of Food Science and Technology, University of West Attica, 122 43 Egaleo, Greece; 5 Department of Biomedical Engineering, University of West Attica, 122 43 Egaleo, Greece; 6 Department of Midwifery, School of Health Sciences, University of West Attica, 122 43 Egaleo, Greece; 7 KidZ Health Castle, Vrije Universiteit Brussel (VUB), UZ Brussel, 1090 Brussels, Belgium

**Keywords:** neonates, formula feeding, breastfeeding, urine metabolomics, synbiotics, NMR spectroscopy, gut microbiota

## Abstract

The aim of this study was to compare the urine metabolic fingerprint of healthy neonates exclusively breastfed with that of neonates fed with a synbiotic-enriched formula (Rontamil^®^ Complete 1) at four time points (the 3rd and 15th days of life and the 2nd and 3rd months). The determination of urine metabolic fingerprint was performed using NMR metabolomics. Multivariate data analyses were performed with SIMCA-P 15.0 software and R language. Non-distinct profiles for both groups (breastfeeding and synbiotic formula) for the two first time points (3rd and 15th days of life) were detected, whereas after the 2nd month of life, a discrimination trend was observed between the two groups, which was further confirmed at the 3rd month of life. A clear discrimination of the synbiotic formula samples was evident when comparing the metabolites taken in the first days of life (3rd day) with those taken in the 2nd and 3rd months of life. In both cases, OPLS-DA models explained more than 75% of the metabolic variance. Non-distinct metabolomic profiles were obtained between breastfed and synbiotic-formula-fed neonates up to the 15th day of life. Discrimination trends were observed only after the 2nd month of the study, which could be attributed to breastfeeding variations and the consequent dynamic profile of urine metabolites compared to the stable ingredients of the synbiotic formula.

## 1. Introduction

Nutritional strategies during the early stages of infant development are considered a pivotal factor in regulating infant metabolism and gut immune system function, thus determining the contingent development of adult metabolic syndromes, i.e., obesity, insulin resistance, gastrointestinal diseases and hypertension [1,2].

Milk is the first food that is introduced into the gastrointestinal (GI) tract after birth, and the composition of milk is believed to directly impact gut microbiota and affect infant neurodevelopment [3,4] through the provision of essential nutrients for bacterial proliferation (i.e., carbohydrates, proteins, iron, phosphor, human milk oligosaccharides (HMOs), etc.), immunomodulatory molecules, small amounts of probiotics and microbes that are capable of colonizing infants [5,6].

Human breast milk (HBM) is acknowledged as the ‘gold standard’ for early nutrition (up to 6 months) and the ‘natural fuel’ for infant neurodevelopment due to its high content of essential nutrients (i.e., carbohydrates, proteins, iron, phosphor, human milk oligosaccharides (HMOs), etc.) and small amounts of probiotics and commensal bacteria [7,8].

Maternally derived microbial metabolites are transmitted to infants via human milk. According to omics studies, breast milk mediates vertical transfer of microbial communities, such as *Streptococcus thermophilus*, *Staphylococcus epidermidis* and *Bifidobacterium longum*, to the neonatal gut [9,10]. Moreover, omics techniques have identified more diverse but stable GI microbiota in formula-fed infants compared to breastfed infants [11,12,13]. According to the most recent literature, human bacterial colonization starts from fetal life, and maternal microbiota seems to influence the neonatal microbiome before conception, during pregnancy, and pre- and post-delivery. In a recent narrative review by Coscia et al. (2021), the currently identified pre- and perinatal factors highlight the influencing on the neonatal microbiota. The authors point out the type of early neonatal nutrition because maternally microbial metabolites transmitted to the infant via human breast milk seem to potentially impact infant health and developmental programming [14]. Furthermore, Stinson et al. (2022) highlighted the potential for microbial metabolites to program immune or metabolic regulatory functions in breastfeeding infants [15].

The infant’s gut microbial composition increases in number and diversity with age and is highly dynamic in the first 6 months of life. A balanced microbiota composition in the first weeks of life predicts microbiota stability throughout the first years of life. Thus, there seems to be a window of opportunity during early infancy that is essentially influenced by the type of feeding and associated with a healthy microbiota profile [16]. At around 3 years of age, the infant attains a mature, adult-like microbiota in terms of diversity and complexity of composition [4,17,18].

Diet is increasingly recognized as a key environmental factor that can modulate the composition and metabolic function of the gut microbiota. Infants can be breastfed, formula-fed or even mixed-fed; these feeding methods influence the gut microbiota at various levels, thus affecting infant health and potentially resulting in consequences later in life [5]. 

In conclusion, targeted nutritional or environmental interventions and readjustments in obstetrical and neonatal medicine practice, such as vaginal seeding [19,20], microbial environment [21], probiotic supplementation [22,23], prebiotic supplementation [24,25,26,27], synbiotic supplementation [28,29,30], human milk feeding [31,32], specific infant formula feeding [33,34] and human donor milk banks [35,36], may be the best strategies to compensate for early-life microbiota disturbance in later life.

However, the decision to practice exclusive or partial breastfeeding is based upon several demographic, socioeconomical, tradition/culture-related and personal criteria [37,38]. In cases of breastfeeding cessation, infant formulae are supplemented as an alternative nutritional approach. The fundamental nutritional components of the most artificial formulae are carbohydrates, water, fats, proteins (casein, whey proteins), minerals, vitamins and nucleotides [39].

Up-to-date advents regarding formula milk composition amend at a rapid pace, focusing on the development of ‘humanized’ formulae that resemble maternal milk, the composition of which is not static but extremely dynamic [40]. Recent studies confirmed that the populations of microbial flora [7,41] and infants’ metabolic fingerprint [2,42] markedly shift in breast- and formula-fed infants. Current trends in infant formulae point to a reduction in formula protein content, which is considered responsible for increasing the risk of childhood obesity, as well as the incorporation of (a) probiotics and (b) lipid constituents (phospholipids, sphingolipids and glycoproteins) or non-human oligosaccharides (galacto-oligosaccharides (GOS) and fructo-oligosaccharides (FOS)) with well-established prebiotic effects [41]. Because prebiotics selectively facilitate the introduction and colonization of the gut by the ingested probiotic communities present in formula, the supplementation of a combined mix of probiotics and prebiotics, called ‘synbiotics’, is recommended [7].

Modification of the gut microbiota by administration of synbiotics, a mixture of prebiotic and probiotic components, might offer a novel and cost-effective strategy in an attempt to reduce the risk of viral infections [43], to restore the delayed colonization of *Bifidobacterium* spp. in C-section-delivered babies [44], to prevent allergies [28,45], to increase the total antioxidant capacity levels in breast milk [29], to prevent weight loss in lactating mothers and to increase weight gain in infants [30].

Non-invasively collected urine and fecal samples are considered the most suitable biological substrates for deciphering the relationship between diet and nutritionally driven metabolic changes in groups of breastfed (BF) and formula-fed (FF) newborns, as they represent the final points of gut microbial metabolism occurring inside human intestinal tract (feces) and of the excreted metabolites produced by the host (urine) [46]. Apart from reflecting nutritional metabolic changes, NMR urine metabolomics progressively find their place as a diagnostic tool for neonatal morbidities involving renal function and food intolerance, namely acute kidney injury (AKI) and necrotizing enterocolitis (NEC), respectively [47,48]. Metabolites of the biological samples under investigation are analyzed by implementing high-throughput, high-sensitivity and reproducible methodologies, such as GC-FID-, LC-MS- or NMR-targeted and/or untargeted metabolomics to holistically identify biomarkers related to different infant nutritional strategies [46].

In the present study, we aspired to determine whether the urine metabolome may act as a functional representative of the gut microbiome as modified by two feeding methods. Therefore, we implemented NMR-based metabolomics with the aim of investigating the effect of exclusive feeding with a synbiotic formula enriched with probiotic *Bifidobacterium animalis (strain BB12)*, as well as prebiotic FOS and polyunsaturated fatty acids (PUFA), such as arachidonic acid (AA), docosahexaenoic acid (DHA) and nucleotides, in comparison with exclusive breastfeeding in terms of any possible correlation with the urine metabolic fingerprint.

## 2. Results

### 2.1. Metabolite Identification

A typical standard ^1^H NMR spectrum of neonate urine with annotations on the identified metabolites is depicted in Supplementary Materials, Figure S1.

### 2.2. Metabolic Enquiries

Several nutritional studies confirmed that the metabolic signature of infants following different feeding practices is strongly associated with their diet [5,49]. Therefore, our first enquiry was directed toward the observance of any metabolic trends between samples of exclusively breastfed (BF) or formula-fed (FF) neonates and infants, identifying signature metabolites that can be related to each group.

**Figure 1:**
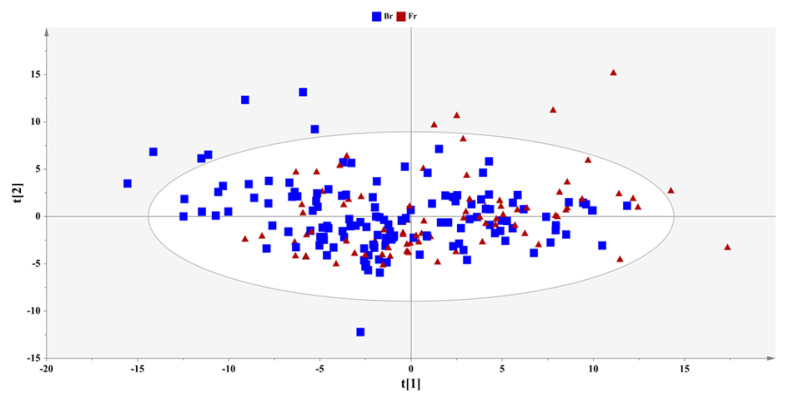
PCA-X model of the complete set of urine samples; A = 2, N = 225, R^2^X(cum) = 0.35, Q^2^(cum) = 0.23, pareto scaling, 95% confidence level (breastfed samples: squares; formula-fed samples: triangles).

A PCA model with two components was computed in the total set of samples to provide an overview and elucidate trends of the sample clustering (Figure 1). Ιn the extracted PCA model, none of the urine samples with either type of feeding (breastfeeding or formula) exhibited any clear clustering trends.

In the subsequent step, supervised analysis was implemented in the overlapping groups to possibly resolve the metabolic variation. Specifically, an OPLS-DA model was computed, including the breast- and formula-fed samples (Supplementary Materials, Figure S2). This model was validated by extracting an ROC curve and permutation testing (Supplementary Materials, Figure S3). In accordance with the AUC for each class, any value above 0.75 was considered acceptable, and the model was considered capable of distinguishing between classes.

**Figure 2:**
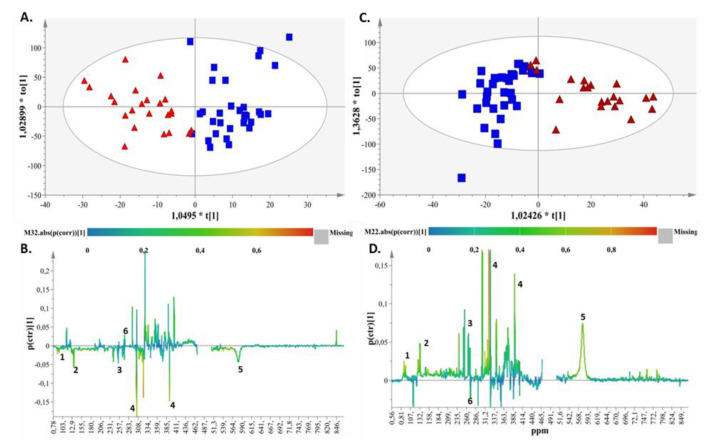
(**A**) OPLS-DA score plot for month 2 for breast- and formula-fed samples, with A = 1 + 1 + 0, N = 56, R^2^X(cum) = 0.57, R^2^Y(cum) = 0.75 and Q^2^(cum) = 0.58 for pareto scaling and a 95% confidence level of *p*-value =3.42 × 10^−10^ (breastfed samples: blue squares; formula-fed samples: red triangles). (**B**) S-line plot demonstrating the metabolites responsible for discrimination (1. bile acids, 2. methyl succinate, 3. citric acid, 4. creatinine, 5. urea, 6. Dimethylamine). (**C**) (a) OPLS-DA scores plot for month 3 for breast- and formula-fed samples, with A = 1 + 1 + 0, N = 54, R2X(cum) = 0.54, R2Y(cum) = 0.72 and Q2(cum) = 0.58 for pareto scaling and a 95% confidence level of *p*-value = 6.66 × 10^−10^ (breastfed samples: squares; formula-fed samples: triangles). (**D**) S-line plot demonstrating the metabolites responsible for discrimination (1. bile acids, 2. methyl succinate, 3. citric acid, 4. creatinine, 5. urea, 6. Dimethylamine).

Using supervised analysis, we attempted to uncover putative differences in the breastfed (BF) versus the formula-fed groups (FF) by comparing the samples at each time point (3rd and 15th days, 2nd and 3rd months). The succinct criteria for model validity and reproducibility regarding the values of R^2^ and Q^2^ (R^2^ values − Q^2^ values > 0.3 and Q^2^ values > 0.5) for the models of the first 2 time points of the study (Supplementary Materials, Figure S4) were not met. On the other hand, the models of the 3rd and 4th sampling points (2nd and 3rd month) presented with high values of goodness of fit and predictability (Supplementary Materials, Table S1). Therefore, the two feeding types presented a significant difference in trend after the 2nd month of life. Specifically, the two groups (BF vs. FF) were clearly discriminated along the first component at 2 and 3 months according to the produced OPLS-DA models (Figure 2A and Figure 2C, respectively). The metabolic profiles of the breastfed and synbiotic-formula-fed samples presented with an almost identical pattern at 2 and 3 months.

The key metabolites related to the FF group according to the corresponding S-line plots (Figure 2B,D) for both time points (2nd and 3rd months) were methyl succinate, citric acid, creatinine, urea and bile acids. Dimethylamine emerged as the discriminant metabolite of the BF group at the same time points (Figure 2B,D).

Finally, we attempted to frame the evolution of metabolites in relation to the four time points for each of the two groups (BF vs. FF). To this end, supervised models were extracted for all sample groups between time points from day 3 to month 3 (Supplementary Materials, Tables S2 and S3).

**Figure 3:**
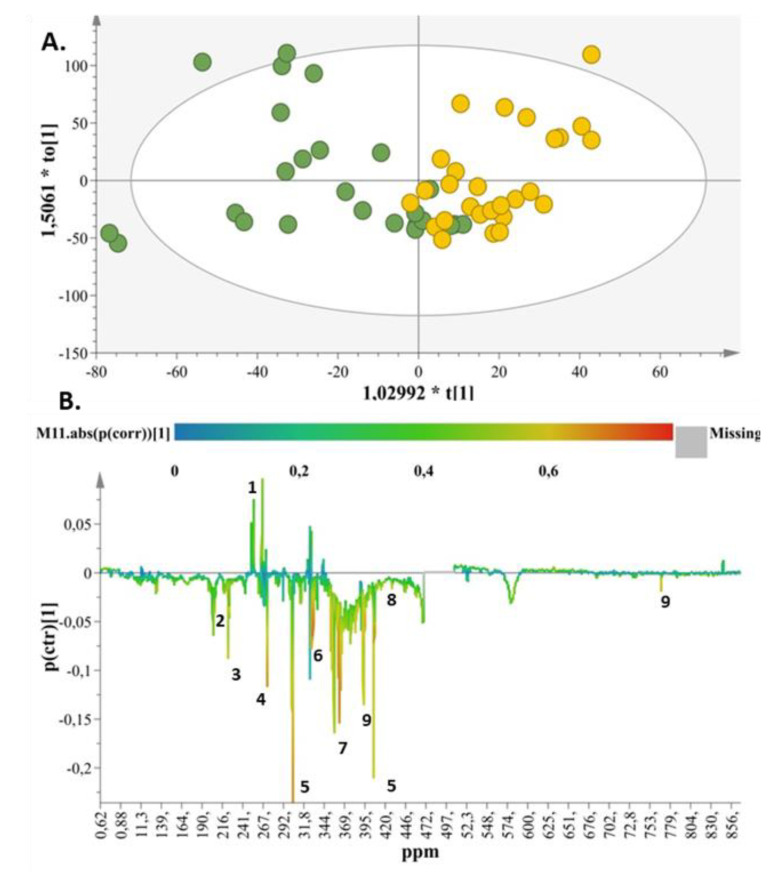
(**A**) OPLS-DA score plot for breastfed samples (day 3 (green circles) vs. month 3 (yellow circles)), with A = 1 + 1 + 0, N = 54, R2X(cum) = 0.57, R2Y(cum) = 0.55, and Q2(cum) = 0.41 for pareto scaling and a 95% confidence level of *p*-value = 0.000139123. (**B**) S-line plot demonstrating the metabolites responsible for discrimination (1. citric acid, 2. glutamine, 3. acetoacetate, 4. dimethylamine, 5. creatinine, 6. betaine, 7. taurine, 8. threonine, 9. Hippurate).

Separation between breastfed samples on collected on the 3rd and 15th days could not be framed in a validated OPLS-DA model (Supplementary Materials, Figure S5), but discrimination was apparent within the same group of BF samples between the 3rd day and the 2nd month (Supplementary Materials, Figure S6), as well as between the 3rd day and the 3rd month (Figure 3A).

Interestingly, the OPLS-DA models of the two different time frames (3rd day versus 2nd and 3rd month) in the BF group and their corresponding S-line plots (Figure 3B and Figure S6) identified glutamine, acetoacetate, dimethylamine, creatinine, betaine, taurine, threonine and hippurate as the key metabolites in the day 3 samples, whereas a high concentration of citric acid characterized the samples collected in the 2nd and 3rd months. Similarly, when comparing 15th day to the 3rd month with respect to breastfed samples (Figure S7), the produced OPLS-DA model provided similar results to those obtained with the previous comparison results between the 3rd day and 3rd month (Figure 3A).

**Figure 4:**
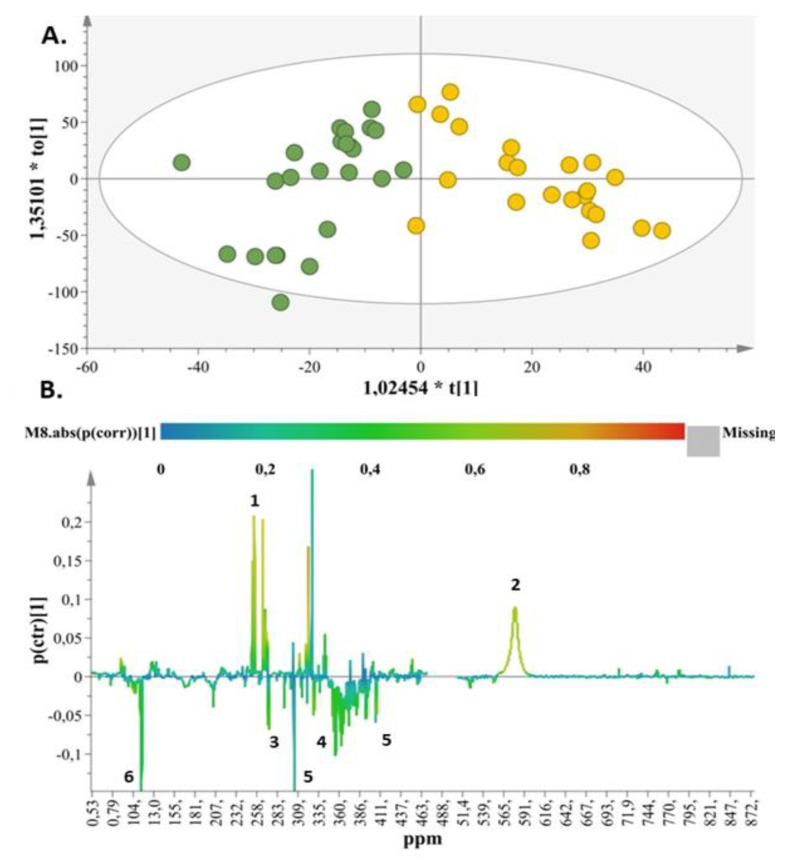
(**A**) OPLS-DA score plot for formula-fed samples (day 3 (green circles) vs. month 3 (yellow circles)), with A = 1 + 1 + 0, N = 48, R^2^X(cum) = 0.51, R^2^Y(cum) = 0.75, and Q^2^(cum) = 0.67 for pareto scaling and a 95% confidence level of *p*-value = 7.33 × 10^−12^. (**B**) S-line plot demonstrating the metabolites responsible for discrimination (1. citric acid, 2. urea, 3. dimethylamine, 4. taurine, 5. creatinine, 6. methyl succinate).

With respect to the formula-fed samples, OPLS-DA models comparing the 3rd day to the 2nd (Figure S8) and 3rd months (Figure 4A) were produced. These comparisons facilitated the interpretation and evaluation of the results between these time frames, as discrimination was not apparent when comparing samples between the 3rd and 15th day (Figure S5). Specifically, in the OPLS-DA model (Figure 4A) the two groups (3rd day vs. 3rd month) are clearly separated. The S-line plot (Figure 4B) correlates citric acid and urea to the samples of the 3rd month and methyl succinate, dimethylamine, creatinine and taurine to the samples of the 3rd day. The same metabolic pattern was observed in the case of formula-fed samples between the 3rd day and 2nd month (Figure S8). Similar results were acquired with the extracted OPLS-DA model by comparing 15th day to 3rd month for the formula-fed samples (Figure S9).

Finally, the use of validation steps (*p* < 0.05, permutation testing and ROC curves) confirmed that the results of all OPLS-DA models were unbiased and reliable, as described in the Supplementary Materials (Figures S10–S15).

### 2.3. Metabolite Pathway Analysis

**Figure 5:**
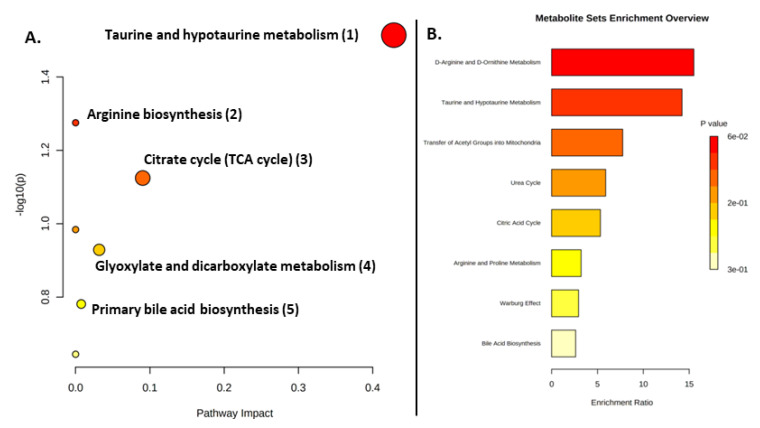
Summary of pathway analysis in the FF group. (**A**) All matched pathways are displayed as circles; the color and size of each circle correspond to *p*-value and pathway impact value, respectively. (**B**) Bar chart of the enrichment analysis results.

Metabolite pathway analysis (MetPA) was performed using Metaboanalyst 5.0 for the metabolites exhibiting an AUROC  >  0.7 in order to delineate metabolic differences for each feeding method. In the context of pathway analysis, tested whether compounds involved in a particular pathway were enriched compared to random hits. The results in the formula-fed group based on the KEGG database demonstrated alterations in taurine and hypotaurine metabolism, arginine biosynthesis, citric acid cycle, alanine, aspartate and glutamate metabolism, glyoxylate and dicarboxylate metabolism and primary bile acid biosynthesis (Figure 5).

Our results pinpointed taurine and hypotaurine metabolism, among other pathways, as being associated with the BF group. In our case, this pathway was significantly upregulated in the FF group relative to the BF group, mainly in relation to the metabolic pathways of fat and protein. This could be explained by the presence of more polyunsaturated fatty acids in infant formula that resembles breast milk, contributing to the digestion and absorption processes [50].

The FF group was also characterized by the citric acid cycle (TCA), a common metabolic pathway for sugars, lipids and amino acids in the mitochondria. On the other hand, pathway analysis using significantly altered metabolites in the breastfed group revealed alterations in alanine, aspartate and glutamate metabolism; glyoxylate and dicarboxylate metabolism; glycine, serine and threonine metabolism; synthesis and degradation of ketone bodies; aminoacyl-tRNA biosynthesis; D-glutamine and D-glutamate metabolism; nitrogen metabolism; valine, leucine and isoleucine biosynthesis; and taurine and hypotaurine metabolism, as presented in Figure 5. The alterations in metabolic pathways resulting from the investigated feeding methods characterize the dynamic relationship between the gut microbiome and the gut metabolome in early life. Although our analyses revealed significant metabolites, reflecting the impact of the gut microbiota on the metabolome, most metabolites were not predictable when evaluated across pathway and enrichment analysis.

## 3. Discussion

Current knowledge confirms time-dependent changes in the metabolic fingerprints and in the biochemical pathways of newborns [51]. Several nutritional studies have confirmed that the metabolic signature of infants following different feeding practices is strongly associated with their diet [5,49]. Such findings verify the hypothesis that in early life, because infants only consume either breast milk or formula, the microbiome participates more actively in metabolic activity than in later life, as infants rely more on microbes to breakdown complex nutrients [52]. Because milk is the first food introduced into the GI tract after birth, it is important to discuss the discriminant metabolites associated with the two investigated feeding practices in newborns at different time points.

The metabolic changes in urine from the complete sample set did not exhibit any clustering trends, as shown in Figure 1. Regarding the evolution of metabolites in relation to the time frame of the study, a comparison between the 3rd and 15th day did not provide any validated models for either feeding type, which suggests that a 2-week period is too short to mirror the impact of feeding in urine metabolic profiling. In accordance with previous reports, the diversity in the microbiota is consistently transmuted with the onset of breastfeeding; thus, variations in the metabolome can probably be framed as a distinct metabolic pattern after the first month of feeding [53].

Clustering between breast- and formula-fed samples was observed after the third time point, i.e., the second month of the study. As shown in Figure 2B,D, for both time points (2nd and 3rd months), the key metabolites related to the FF group were methyl succinate, citric acid, creatinine, urea and bile acids, whereas dimethylamine emerged as the discriminant metabolite in the BF group.

In particular, primary and conjugated bile acids, key regulators in the formation of gut microecology by participating in bacterial metabolism, were detected in high amounts in healthy neonates [54]. Secretion of bile acids (mainly sulfated) is elicited by the presence of human milk lipids and is indirectly associated with increased presence of *Veillonella*, *Bacteroides*, *Clostridium*, *Enterobacteriaceae* and *Streptococcus* taxa in BF newborns [55,56]. According to in vivo studies in weanling rats, higher concentrations of secondary bile acids (i.e., deoxycholic acid) were determined in the formula fed group. In line with our results, secondary bile acids may be representative metabolic products of probiotics, such as *Bifidobacteria*, or bacterial communities, such as *Clostridia* and *Firmicutes*, in FF infants from 1st to 3rd month of life [56].

In line with our findings (Figure 3 and Figure 4), taurine, a sulfur-containing amino acid that participates in the synthesis of bile acids and is received through human milk or infant formula, showed a decreasing urinary excretion trend 3 months after birth [57].

Variations in the levels of citric acid produced during the tricarboxylic acid (TCA) cycle are affected by factors related to gestational age, lactation, height, weight, growth rate and energy demands of the newborn [58].

In the present study, citric acid was found to share a common metabolic fate through time, as its levels increased with age in both groups (3rd month compared to the 3rd day), as supported by Figure 3 and Figure 4. In accordance with our results, a previous clinical intervention with subjects receiving bovine or donkey breast milk indicated citric acid as a maturation biomarker for this group [52,58]. However, citric acid is also a key compound of the metabolic excretion profile during the first days (1st to 7th day) of life in BF infants [1,52,59]. Notably, the urine fingerprint of FF neonates in our study highlighted citric acid as a discriminatory metabolite of this feeding type after the second month of the study.

Urea is considered the most important source of nitrogen, and its presence in the urine metabolome of newborns is anticipated. A strong correlation of urea with the BF group was closely associated with the modification of their intestinal microbiome and the increase in commensal bacterial populations, such as *Bifidobacteria* [60]. Nonetheless, in the case of the FF group, the consistent intake of protein-enriched infant formula for a certain period of time (3 months in our study) normally leads to increased levels of nitrogen metabolites, such as urea and creatine, which are related to the protein degradation pathways (Figure 5) [42,61,62]. In contrast, due to its high lipid content, breastfeeding seems to favor fatty acid catabolism rather than protein catabolism [62]; therefore, reduced concentrations of urea months after birth are can be attributed to the reduced yet more efficient use of proteins in nourishing infants [63].

Regarding the assignment of methyl-succinate as a signature marker for the synbiotic formula group (Figure 2), the results of the present study are in line with the findings of other research groups [64]. The increase in this metabolite in FF newborns is driven by the catabolic pathways of amino acids, such as isoleucine, which is found in high-protein infant formulae. Moreover, recent studies of fecal cultures of healthy 2-month-old neonates revealed the interrelation of succinate compounds with the establishment of commensal *Enterobacteriaceae* communities in the FF infant gut [65]. Apart from the feeding mode, the concentrations of methyl-succinate has been shown to significantly depend on the age of the infant [64].

Our findings indicate creatinine as a discriminant compound, as it was found in higher levels in the FF group than in the BF group after 2 months of life (Figure 2), which may be closely related to the rapid degradation rate of proteins in protein-rich formulae [62,66]. The decreased creatinine levels in both FF and BF newborns in the 3rd month compared to the 3rd day of the study (Figure 3 and Figure 4) are in agreement with other published scientific data [64,67]. As indicated by the plasma metabolic signature of BF and FF infants, creatinine showed a decreasing tendency over the course of the infant’s lifespan [67]. An in vivo study in monkeys also reported that creatinine levels were elevated in the first days of life [68].

Special attention should be paid to dimethylamine, which plays an essential role in the clustering of FF vs. BF urine samples (Figure 2). Recent findings also highlighted dimethylamine as a discriminant microbial metabolite of BF newborns compared to FF infants [66]. Specifically, dimethylamine is a secondary amine found in excess in urine samples as a result of ADMA hydrolysis, an endogenous inhibitor of nitric acid biosynthesis, or due to TMAO oxidation, a compound found in food matrices that is also produced in human gut microflora as choline metabolite [69]. Finally, dimethylamine is considered an important bacterial byproduct of amino acid pathways [42].

In relation to gut microflora, dimethylamine was found to be positively associated with *Proteobacteria*, whereas it was negatively correlated with *Actinobacteria* (i.e., *Bifidobacterium*) [70]. However, the continual reshaping of infants’ gut microbial populations in the first months of postnatal life normally results in significant variations at the level of dimethylamine in association with both investigated feeding approaches at various sampling points [71]. Most importantly, dimethylamine is an early microflora metabolite of microbiota–host reciprocal interactions; thus, its levels could increase during the first days of breast or formula feeding [70]. This phenomenon was apparent in both the BF and FF models in our study between the 3rd day and the 3rd month (Figure 3 and Figure 4).

The lower number of differentiating metabolites in the BF group in the 3rd month compared to 3rd day (Figure 3 and Figure S6) may be ascribed to the gradual colonization/establishment of microbial communities and the maturation of the overall infant metabolism that takes place between the 3rd day and the 3rd month [72], which, in our study, was mirrored by the presence of one discriminant metabolite (citric acid) in urine at in the 2nd and 3rd months compared to that on the 3rd day.

On the other hand, incomplete kidney maturation and high choline intake appeared to be responsible for the increased production of betaine, a product of choline oxidation. In neonatal urine samples reported in previous study, betaine was increased progressively from the 3rd day to 3rd month before exhibiting a sharp decrease in infants at the age of 6 months [51,67]. Increased amounts of betaine on the 3rd day of life in the BF group (Figure 3) may also be related to the elevated levels of TMAO from 3rd day to the 3rd; because TMAO is a gut-generated metabolite, its concentration is strongly associated with the ongoing maturation of intestinal microflora (i.e., increase in *Akkermansia* abundance) [73,74,75]. A previous study investigating healthy term infants in relation to their feeding regimen using capillary electrophoresis time-of-flight mass spectrometry (CE-TOF/MS) revealed that betaine, together with other choline metabolites (N,N-dimethylglycine and sarcosine), differentiated the BF group from the FF group only at the age of 1 month, at which time level of excretion of choline metabolites was higher in BF infants than FF infants [76]. Although betaine was identified as a discriminant metabolite in BF infants in our study, as was increased on the 3rd day compared to 3rd month (Figure 3), it was not found to discriminate between formula- and breastfed infants.

Finally, threonine was observed in higher concentrations at the beginning of the study compared to the 3rd month in the BF group (Figure 3). A reducing trend in threonine levels during the postnatal period was also reported by Lönnerdalet al. (2016) in the plasma of 4- and 6-months-old infants [77]. The excretion of urinary threonine was also decreased after the first month of life in BF newborns [1]. 

## 4. Materials and Methods

### 4.1. Sample Collection

Study subjects were allocated into 2 groups: one group exclusively breastfed (BF) and the other group receiving a synbiotic formula (Rontamil^®^ Complete 1—*Bifidobacterium animalis* 1.00 × 10^7^ cfu/g, FOS 0.52 g/100 kcal, Rontamil, Zug, Switzerland) (FF). In the FF group, neonates were exclusively breastfed prior to enrolment. In the case of medical indication or parental wish for formula introduction along with breastfeeding, the neonates received Rontamil^®^ Complete 1 infant formula. The average nutritional composition per 100 mL of human breast milk and formula (Rontamil^®^ Complete 1) is presented in Table S4 [78,79]. Urine samples from 72 neonates (36 from each group) were collected at 4 time points (3rd and 15th days of life, 2nd and 3rd months). Healthy, full-term singleton neonates born either by vaginal delivery or caesarian section with an Apgar score > 7 at the 1st and 5th minute, not requiring any intervention at birth and exhibiting normal intrauterine growth (BW 10–89th centile) were eligible for the study. WHO growth charts were used to calculate the percentile of birth body weight. Exclusion criteria included intrauterine growth restriction, positive family history of cow’s milk allergy, admission to neonatal intensive care unit and intra/ postpartum maternal or neonatal antibiotic use. The mean enrolled postnatal age was 38 weeks and 5 days. The birth weight (mean, SD), together with the ratios of sex and delivery mode, are provided for both groups in Supplementary Materials, Table S5.

Approval (No.ΕΕ-2/15/31-01-2017) from Aretaieio Hospital Review Board, along with the Ethics committee, as well as approval (No.AΔA: ΩΔΖΧ46906Ψ-450/09-03-2017) from Nikaia General Hospital “Agios Panteleimon”, were received prior to initiation of the study. All experiments were performed in accordance with existing guidelines and regulations. Signed informed consent was obtained from the participating mothers prior to enrolment. The clinical study was registered with ClinicalTrials.gov under the title, “Metabolomic Profile of Urine Samples from Neonates fed with breastmilk and infant formula enriched with synbiotics” with registration number NCT03320837.

### 4.2. Metabolomic Analysis

#### 4.2.1. Sample Preparation

The samples were thawed at room temperature 60 min before performing the NMR experiments. Samples were centrifuged (10000 g, 4 °C, 10 min), and 450 µL of urine was mixed with 150 µL of a 1.5 M phosphate buffer (pH 7.4) in D_2_O containing 0.1% sodium trimethylsilyl propionate (TSP) and sodium azide (NaN_3_, 2 mM); then, samples were transferred to 5 mm NMR tubes [80].

#### 4.2.2. NMR Measurements and Data Processing

All NMR spectra were acquired on a Varian-600 MHz NMR spectrometer (Varian, Palo Alto, CA, USA) equipped with a triple-resonance probe {HCN}. One-dimensional 1H-NMR spectra were collected at 25 °C with the 1D NOESYPRESAT pulse sequence for solvent signal suppression.

All 1H NMR spectra were phase- and baseline-corrected using Mnovav.10.1 software (Biotechnology, Gaithersburg, MD, USA). The NMR spectrum of each sample was aligned with reference to the TSP signal at δ 0.00 ppm. The 1H NMR spectra were reduced into buckets of 0.0001 ppm, and the D_2_O region was removed. The spectra were normalized to the standardized area of the reference compound and converted to ASCII format using Mnovav.10.1 software. The ASCII files were imported into MATLAB (R2006a, Mathworks, Inc., Natick, MA, USA, 2006), and all spectra were aligned using the correlation optimized warping (COW) method [81].

#### 4.2.3. Metabolite Identification

2D NMR spectroscopy was utilized to assist in the assignment of metabolites. Specifically, gCOSY, zTOCSY, gHMBCad and gHSQCad were recorded at 25 °C. The acquisition parameters for gCOSY were: spectral width (SW), 7225.4 Hz; t1 increment, 256; acquisition time, 0.150 s; number of scans, 128; 1084 data points; receiver gain, 30; and relaxation delay, 1 s. The acquisition parameters for zTOCSY were set at: spectral width (SW), 7225.4 Hz; t1 increment, 256; number of scans, 128; acquisition time, 0.283 s; 2048 data points; receiver gain, 30; and relaxation delay, 1 s. The acquisition parameters for gHSQCad were: f2 spectral width (SW), 7225.4 Hz; f1 spectral width (SW), 30165.9 Hz; t1 increment, 256; number of scans, 128; acquisition time, 0.150 s; 1084 data points; receiver gain, 30; and relaxation delay, 1 s. The acquisition parameters for gHMBCad were: f2 spectral width (SW), 7225.4 Hz; f1 spectral width (SW), 36199.1 Hz; t1 increment, 256; number of scans, 128; acquisition time, 0.150 s; 1084 data points; receiver gain, 40; and relaxation delay, 1 s.

2D spectra were interpreted with MestReNova v.10.1 software (Mestrelab, A Coruña, Spain). The identification procedure was also assisted by literature data [82,83], a reference metabolite ^1^H NMR database (Chenomx NMR Suite 8.0, Chenomx Inc., Edmonton, AB, Canada) and an in-house, fully automated metabolite identification platform [84].

### 4.3. Statistical Analysis

#### 4.3.1. Post Processing of Spectral Data

Multivariate statistical data analysis was performed using ASICS and the ropls [85] R packages (version 4.1), together with SIMCA-P version 15.0 (Umetrics, Umeå, Sweden).

Spectral data were mean-centered and Pareto-scaled (Par) [86]. Multivariate statistical analysis included principal component analysis (PCA) [87], partial least squares discriminant analysis (PLS-DA) and orthogonal projection to latent structures discriminant analysis (OPLS-DA).The mathematical background and applications of these methods have been extensively discussed in the literature [88,89,90,91,92].

#### 4.3.2. Identification of Important Features and Model Validation for SIMCA-P

Feature selection for the OPLS-DA models was based on variable importance in projection (VIP) scores > 0.7 and P(corr)  >  0.2 to reveal the variables with class-discriminating power. S-line plots were facilitated to pinpoint metabolites that contributed to sample discrimination.

The quality of models (PCA/OPLS-DA) was described by the goodness-of-fit R^2^ (0 ≤ R^2^ ≤ 1) and the predictive ability Q^2^ (0 ≤ Q^2^ ≤ 1) values. R^2^ explains the variation, constituting a quantitative measure of the quality of the mathematical reproduction of the training set data. The overall predictive ability of the model was assessed by the cumulative Q^2^, representing the fraction of the variation of Y that can be predicted by the model, which was extracted according to the internal cross-validation default method of SIMCA-P software, version 14.0 (Umetrics, Malmo, Sweden). Q^2^ is considered a de facto default diagnostic parameter to validate OPLS-DA models in metabolomics. In particular, all OPLS-DA models demonstrated high statistical values (R^2^ > 0.7 and Q^2^ ≥ 0.50), the difference between the goodness-of-fit and the predictive ability always remained below 0.3 (R^2^X(cum) − Q^2^ (cum) < 0.3) and the goodness of fit was never equal to one (R^2^X(cum) ≠ 1). Therefore, because the extracted models abided by these rules, their robustness and predictive response were enhanced, and overfitting was avoided.

Classification models were validated using cross-validation analysis of variance (CV-ANOVA), with a *p*-value < 0.05, as noted in each multivariate statistical model. Furthermore, permutation tests were employed (999 permutations) in order to evaluate whether the specific classification of two classes in a model was significantly better than any other models obtained by randomly permuting the original group attribution. All models were extracted at a confidence level of 95%.

An additional measure of PLS-DA model validity included the extraction of receiver–operator characteristic (ROC) curves to assess the ability of the PLS latent variable Tpred to correctly classify the test set. The area under the ROC (AUROC) was calculated. A perfect discrimination corresponded to an AUROC equal to 1.

#### 4.3.3. Model Validation for R

The package ropls (R) was employed for the implementation of the PCA, the partial least squares-discriminant (PLS-DA) and its orthogonal implementation (OPLS-DA). These techniques are used in to maximize the correlation between two sets of variables by reducing the data to a few latent variables. The orthogonal use of this method provides improved interpretation of variations between the discriminated groups. The evaluation of PLS-DA and OPLS-DA models was based on the goodness-of-fit coefficient (R^2^Y_ and the goodness-of-prediction coefficient (Q^2^Y). The prediction was estimated by 7-fold cross validation, and the robustness of the models was measured through permutation testing for 999 iterations. For the PLS models, the optimal number of latent variables used was based on the accumulation of high R^2^ values without model overfitting.

In addition, the ASICS package was employed in order to identify and quantify metabolites from the NMR data. To identify statistically significant variables that affected the two discriminations, a Kruskal–Wallis test [93] was performed, and the *p*-values of multiple tests were corrected with the Benjamini–Hochberg method [94].

#### 4.3.4. Metabolite Pathway Analysis

Metaboanalyst 5.0 was used (http://www.metaboanalyst.ca, accessed on 20 July 2022) for biomarker discovery, classification and pathway mapping. A hypergeometric test using over-representation analysis and pathway topology analysis related these metabolites to metabolic pathways. MetaboAnalyst 5.0 (http://www.metaboanalyst.ca/metaboanalyst/, accessed on 20 July 2022) was used for bioinformatics analysis, and the pathway analysis module based on the KEGG database was applied to reveal relevant differential metabolic pathways.

## 5. Conclusions

We performed a metabolomics analysis utilizing urine samples from a relatively large birth cohort study at four time points and considering two feeding methods (breast feeding and an infant formula rich in synbiotics, polyunsaturated fatty acids and nucleotides). According to established knowledge, we attempted to elucidate alterations in the urine metabolic fingerprint correlating with putative changes in the gut microbiota.

In this context, it was evident that the urine metabolic profiles of BF and synbiotic-enriched FF infants were similar during the two first time points of the study. Elucidation of the evolution of metabolites versus time showed that a 2-week period is too short to mirror the effect of different types of feeding in the urine metabolome. An impact on the metabolic fingerprint was evident after the second month, with citric acid, methyl succinate, urea and bile acids, as well as dimethylamine, as discriminant metabolites. Citric acid shared a common metabolic fate through time, as its levels were increased with age in both groups in the 3rd month compared to the 3rd day. With respect to the FF group, the consistent intake of a protein-enriched infant formula for more than 2 months may have led to increased levels of nitrogen metabolites, such as urea and creatine. The increase in methyl-succinate in FF newborns seems to have been driven by the catabolic pathways of amino acids, such as isoleucine, present in protein-rich infant formulae.

Although the relationship between the gut microbiota and the effect on the metabolome is not well understood, increasing evidence suggests that the infant’s intestinal microbiome plays a key role in metabolism and immune development, with impacts on lifelong health.

In many cases, alterations of metabolites may be attributed to the shaping of the gut microecology over time and by the participation of metabolites in bacterial metabolism. For instance, conjugated bile acids are a key regulator of gut microecology by participating in bacterial metabolism and were correlated with the FF group. Moreover, dimethylamine is an early microflora metabolite of microbiota–host reciprocal interactions; thus, it is present in higher levels in the first days of both in BF and FF infants. Nevertheless, it remains a characteristic metabolite for discrimination between BF and FF infants, even in later stages (month 2). Overall, this study reveals that both investigated feeding types may exert beneficial effects directly or indirectly through alterations in the gut microbiota. However, this study is limited by the use of urine samples as the sole substrate, and further validation is required. The role of metabolomics is also highlighted to provide comparative data regarding the effect of different feeding regimes on infant metabolism. Additionally, despite the short sampling intervals, the metabolic frame at each sample point provides only a snapshot of the constantly changing microbiota ecosystem. Generally, understanding the crosstalk between feeding type, gut microbiota and consequent metabolome will facilitate the development of novel and personalized dietary interventions during early life, which may prove useful to prevent metabolic disorders in later years.

## Data Availability

Not applicable.

## Supplementary Materials

The following supporting information can be downloaded at: https://www.mdpi.com/article/10.3390/ijms231810476/s1.
