# Supplementary material for: Urine Metabolomic Profile of Breast- versus Formula-Fed Neonates Using a Synbiotic-Enriched Formula
Source: Int J Mol Sci. 2022 Sep 9;23(18):10476. doi: 10.3390/ijms231810476 (PMC9499619; doi:10.3390/ijms231810476)
Supplement: Supplementary file 1 [file ijms-23-10476-s001.zip › ijms-1868566-supplementary.pdf]

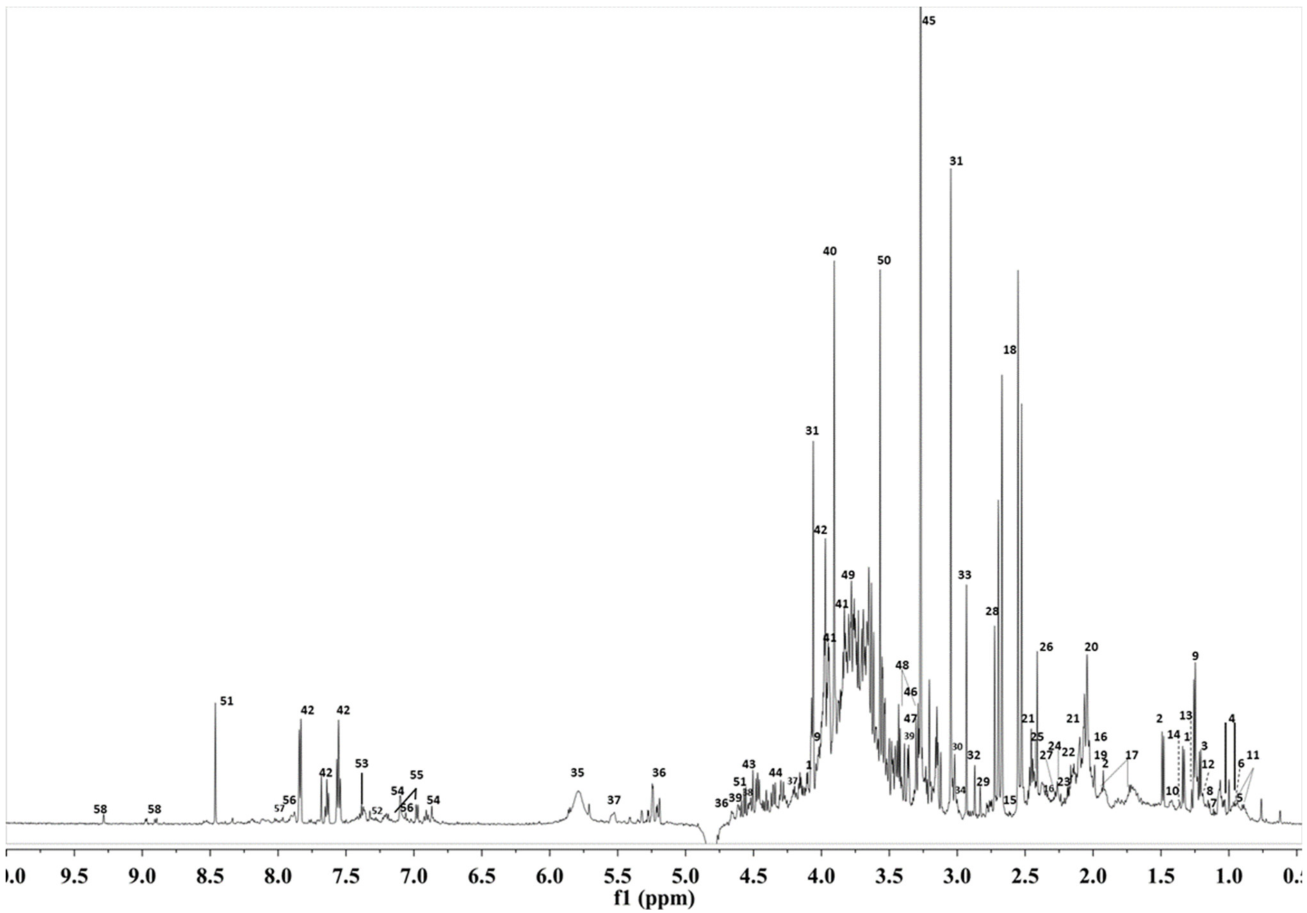

Figure S1. 1H NMR spectra of a urine sample with annotation on the identified metabolites. 1:lactate; 2:alanine; 3:3-amino-isobutyrate; 4:valine; 5:isoleucine; 6:leucine; 7:isobutyrate; 8: succinic acid; 9:L-fucose; 10:lysine; 11:bile acids (2-hydroxybutyrate); 12:3-methyl-2-oxovalerate; 13:3-hydroxyisovalerate; 14:2-hydroxyisobutyrate. 15:β-alanine; 16:2-hydroxyglutarate; 17:lysine; 18:citrate; 19:acetate; 20:N-acetylaspartate; 21:L-glutamine; 22:acetone; 23:aminoadipate; 24:acetoacetate; 25:pyroglutarate; 26:pyruvate; 27:isobutyrate; 28:dimethylamine; 29:methylguanidine; 30:creatine; 31:creatinine; 32:trimethylamine; 33:dimethylglycine; 34: isocitrate; 35:urea; 36:D-glucose; 37:sucrose; 38: L-cystine; 39:gluconate; 40:glycolate; 41:serine; 42:hippurate; 43:trigonelline; 44:threonine; 45:choline; 46:trimethylamine-N-oxide; 47:betaine; 48:taurine; 49:guanidoacetate; 50:glycine; 51:formate; 52:τ-methylhistidine; 53:N-phenylacetylglycine ; 54:phenol; 55:tyrosine; 56:histidine; 57: π-methylhistidine, 58: trigonelline

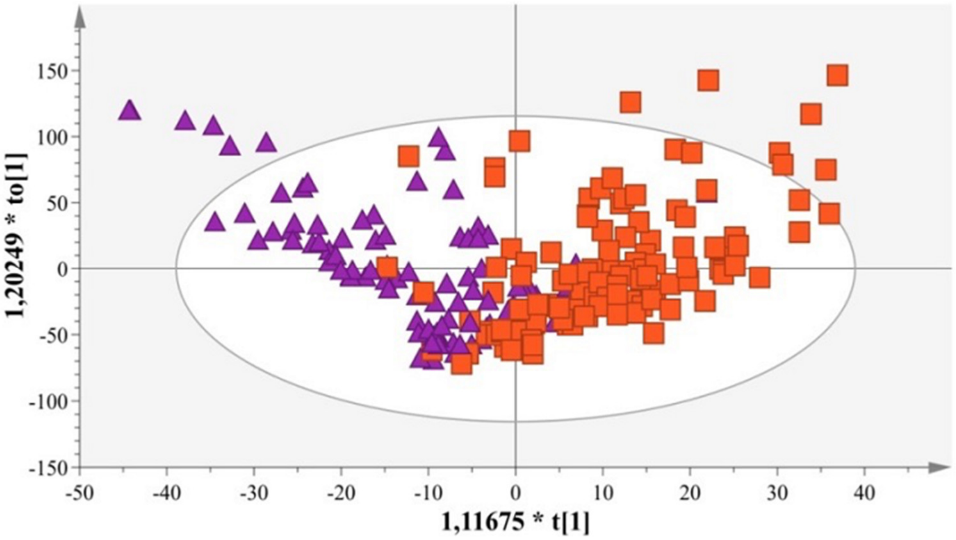

Figure S2. OPLS-DA scores plot (breast vs formula-fed), with A=1+1+0, N=225, R^2X(cum)=0.52, R^2Y(cum)=0.51, Q^2(cum)=0.44 for pareto scaling and 95% confidence level, p-value= 6,27E-33. (breast-fed samples: squares, formula-fed samples: triangles).

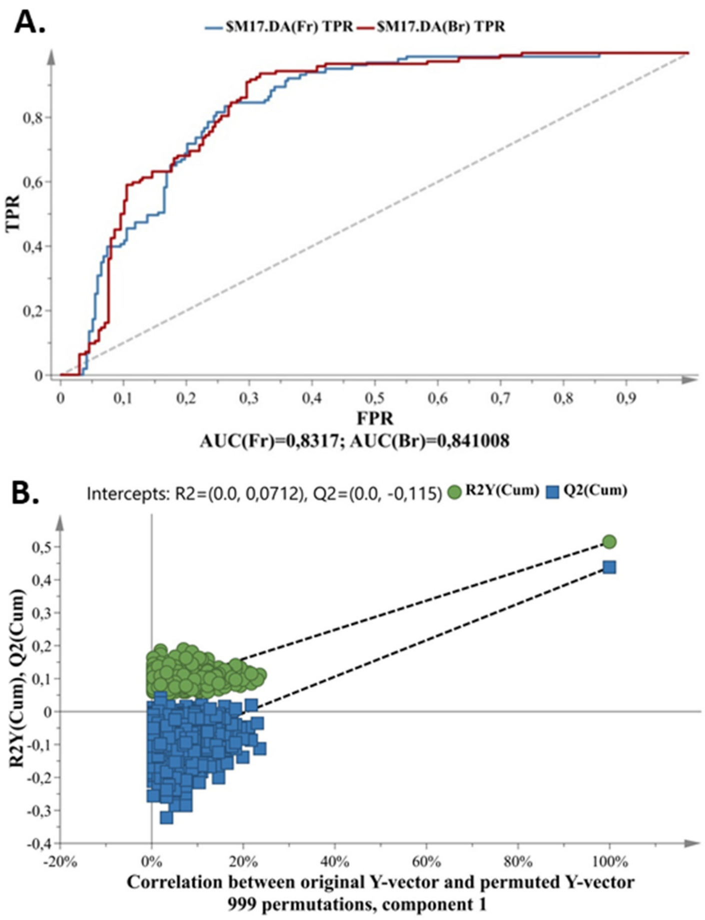

Figure S3. (a) ROC curve AUC (Synbiotic formula) = 0.8317 and AUC(Breastfeeding)=0.841 **(b)** Permutation testing for the OPLS-DA model in Figure S2, Synbiotic formula vs Breastfeeding.

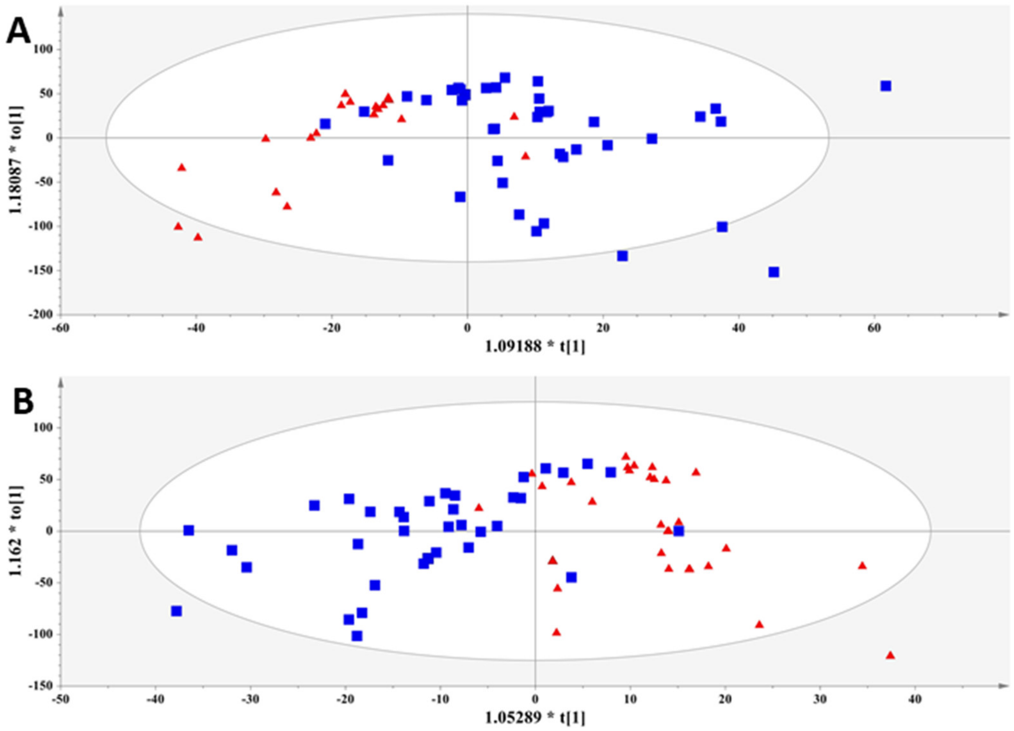

Figure S4. **(a)** OPLS-DA scores plot for day 3 regarding breast- and formula-fed samples, with A=1+1+0, N=64, R^2X(cum)=0.61, R^2Y(cum)=0.46, Q^2(cum)=-0.23 for pareto scaling and 95% confidence level, p-value>0,05. (Breast-fed samples: blue squares, formula-fed samples: red triangles); **(b)** OPLS-DA scores plot for day 15 regarding breast- and formula-fed samples, with A=1+1, N=67, R^2X(cum)=0.61, R^2Y(cum)=0.55, Q^2(cum)=0.17 for pareto scaling and 95% confidence level, p-value>0,05. . (Breast-fed samples: blue squares, formula-fed samples: red triangles)

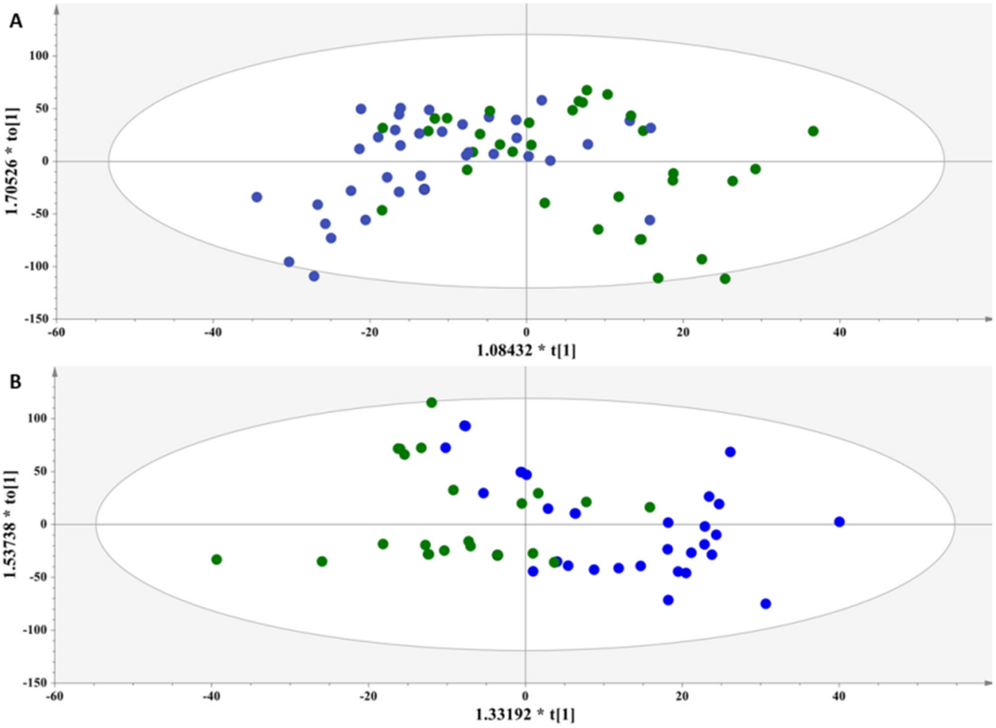

Figure S5. (a) OPLS-DA scores plot for breast-fed samples regarding day 3 and day 15, with A=1+1+0, N=64, R^2X(cum)=0.61, R^2Y(cum)=0.46, Q^2(cum)=-0.23 for pareto scaling and 95% confidence level, p-value>0,05. . (Breast-fed samples: blue squares, formula-fed samples: red triangles); **(b)** OPLS-DA scores plot for formula-fed samples regarding day 3 and day 15, with A=1+1, N=67, R^2X(cum)=0.61, R^2Y(cum)=0.55, Q^2(cum)=0.17 for pareto scaling and 95% confidence level, p-value>0,05. (Breast-fed samples: blue squares, formula-fed samples: red triangles)

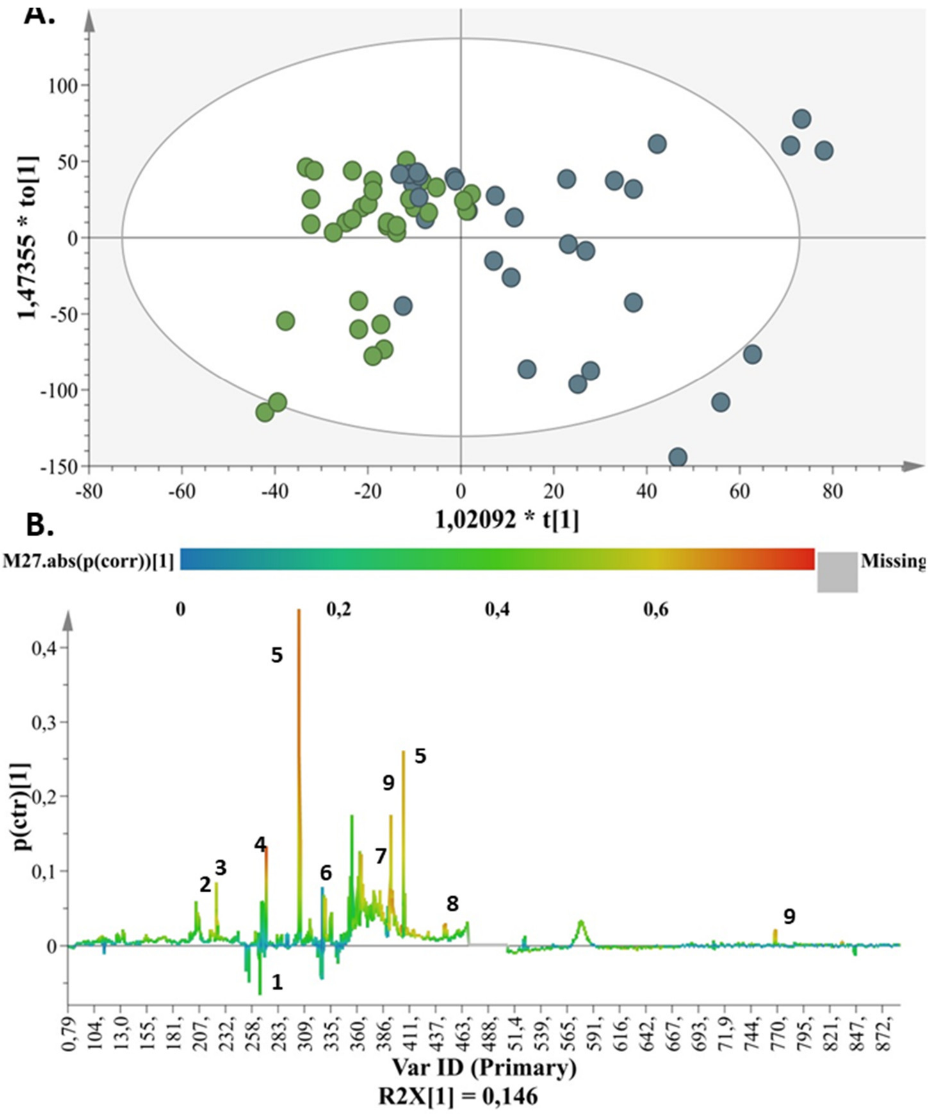

Figure S6. (a) OPLS-DA scores plot for breast-fed samples (day 3 vs month 2), with A=1+1+0, N=64, R^2X(cum)=0,61, R2Y(cum)=0,48, Q^2(cum)=0,33, for pareto scaling and 95% confidence level, p-value= 3,32E-05. (day 3: blue circles, month 2: green circles); **(b)** S-line plot demonstrates the metabolites responsible for discrimination (1. citric acid, 2. glutamine, 3. acetoacetate, 4. dimethylamine, 5. creatinine, 6. betaine, 7. taurine, 8. threonine, 9. hippurate).

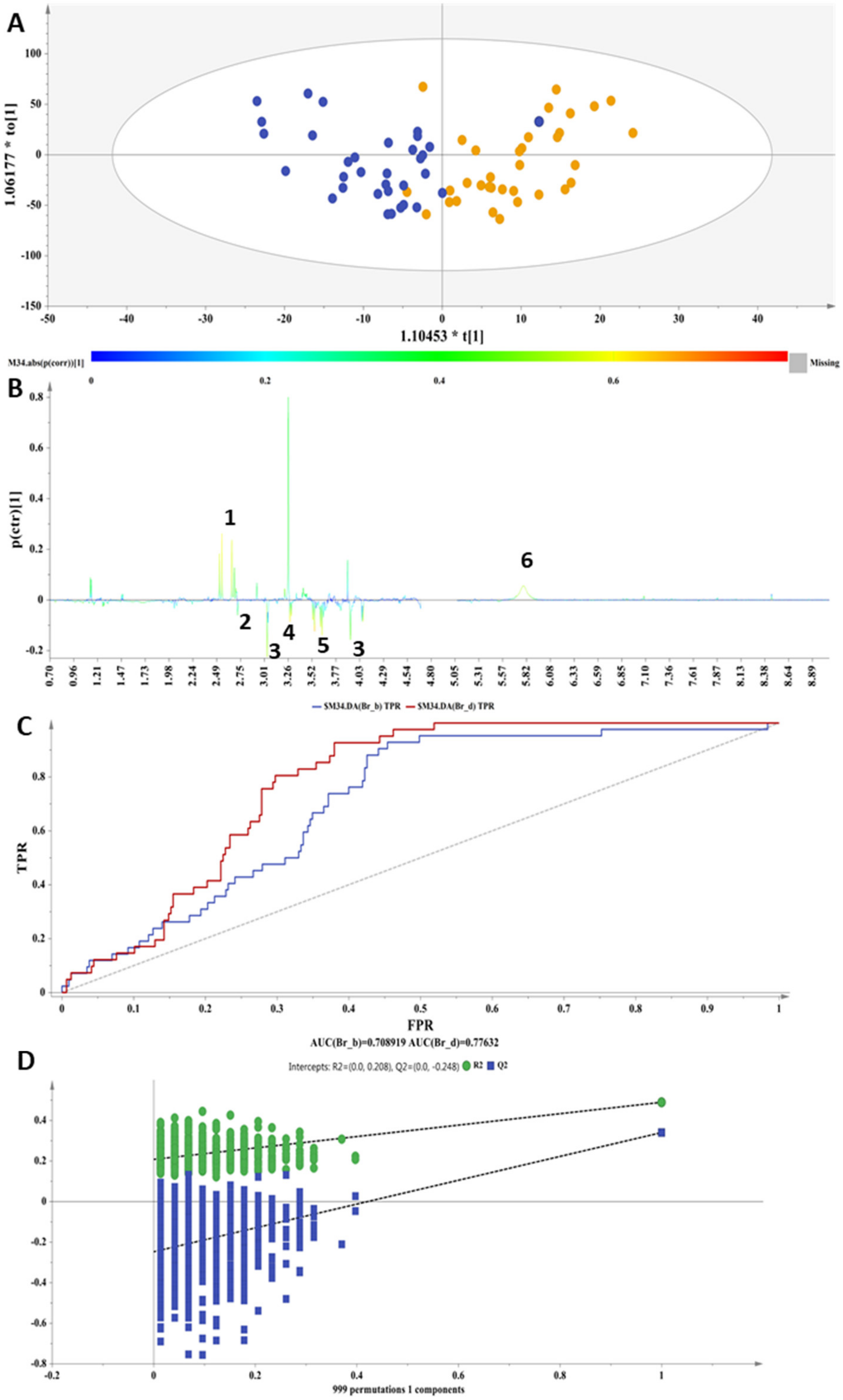

Figure S7. **(a)** OPLS-DA scores plot for breast-fed samples regarding day 15th and month 3, with A=1+1+0, N=67, R^2X(cum)=0.55, R^2Y(cum)=0.49, Q^2(cum)=0.34 for pareto scaling and 95% confidence level, p-value= 9.21236e-006. (yellow squares: month 3, blue squares: day 15); **(b)** S-line plot demonstrates the metabolites responsible for discrimination. 1. citric acid, 2. dimethylamine, 3. Betaine, 4. Creatinine, 5. taurine, 6. urea. **(c)** ROC curve AUC (15th day) = 0.709 and AUC (3rd month) =0.776; **(b)** permutation testing for the OPLS-DA model for breast-fed 15th day vs 3rd month.

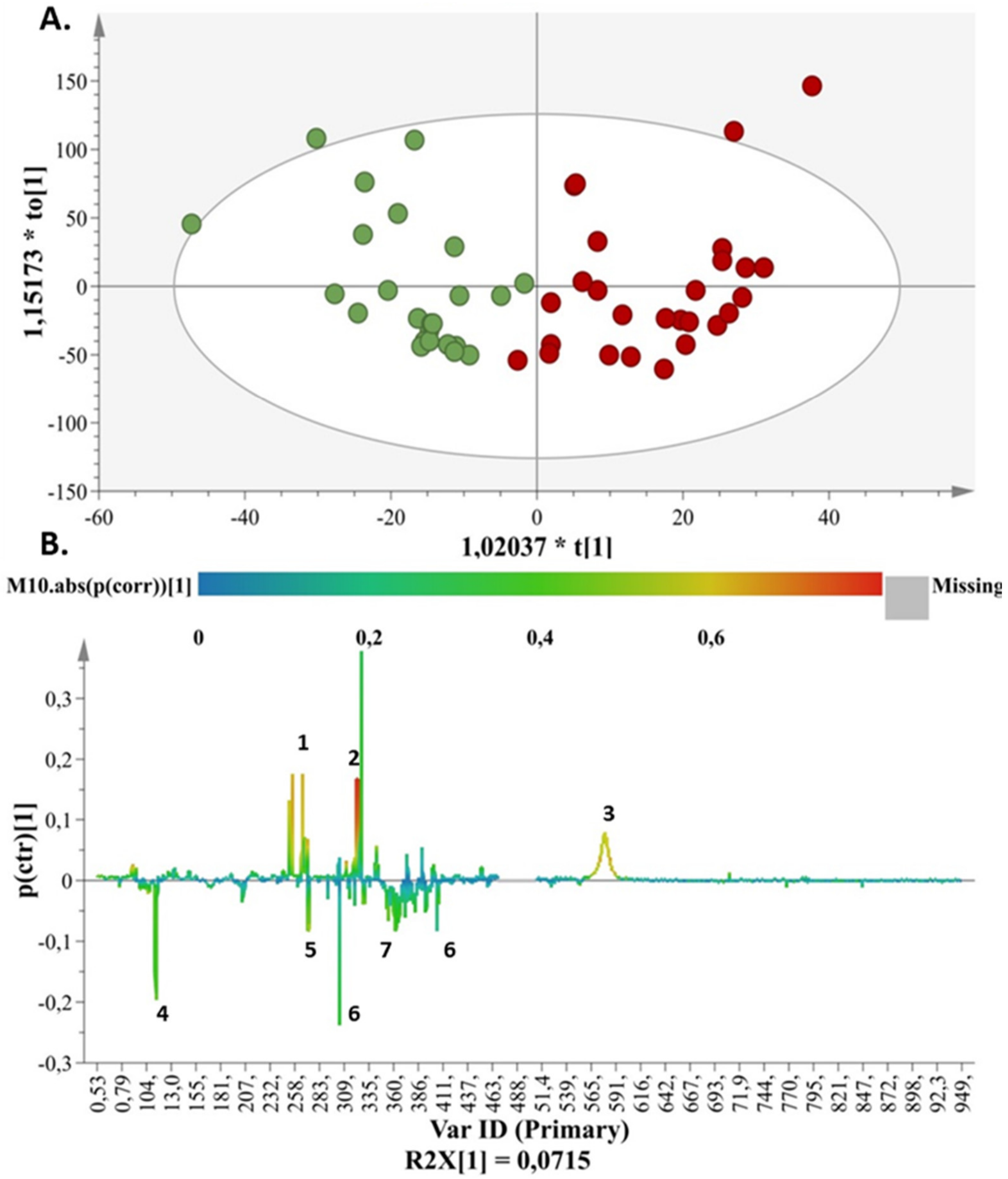

Figure S8. (a) OPLS-DA scores plot for formula-fed samples (day 3 vs month 2), with A=1+1+0, N=53, R^2X(cum)=0.53, R^2Y(cum)=0,75, Q^2(cum)=0.55 for pareto scaling and 95% confidence level, p-value= 7,33E-12, (day 3: green circles, month 2: red circles); **(b)** S-line plot demonstrates the metabolites responsible for discrimination. (1. citric acid, 2. betaine, 3. urea, 4. methyl succinate, 5. dimethylamine, 6. creatinine, 7. Taurine).

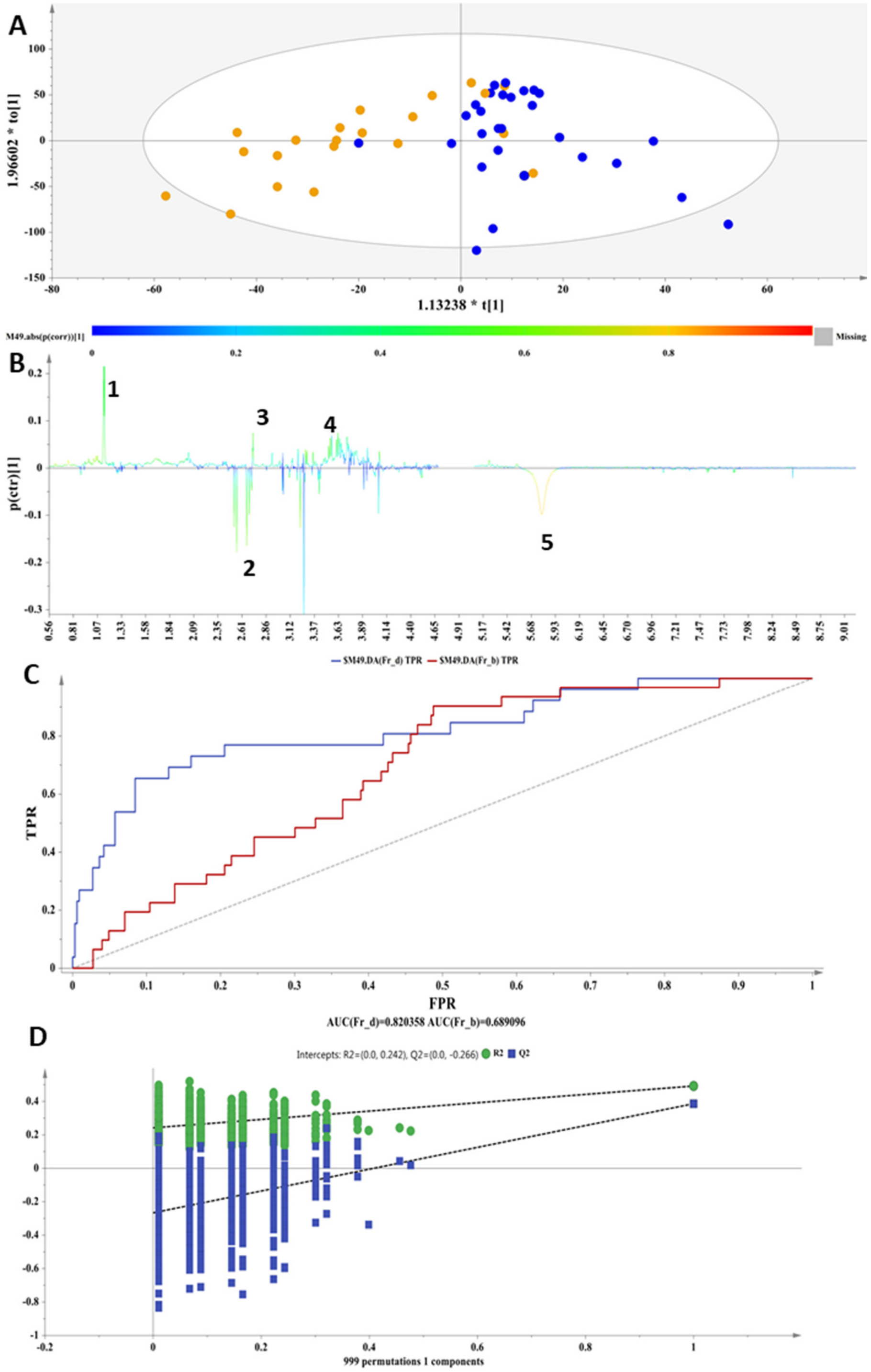

Figure S9. (a) OPLS-DA scores plot for formula-fed samples regarding day 15th and month 3, with A=1+1+0, N=50, $R^2X(cum)=0.59$, $R^2Y(cum)=0.49$, $Q^2(cum)=0.39$ for pareto scaling and 95% confidence level, p-value= 8.3714e-005. (yellow squares: month 3, blue squares: day 15:); **(b)** S-line plot demonstrates the metabolites responsible for discrimination. 1. methyl succinate, 2. citric acid, 3. dimethylamine, 4. Taurine, **(c)** ROC curve AUC (15th day) = 0.689 and AUC (3rd month) =0.820; **(b)** permutation testing for the OPLS-DA model formula-fed 15th day vs 3rd month.

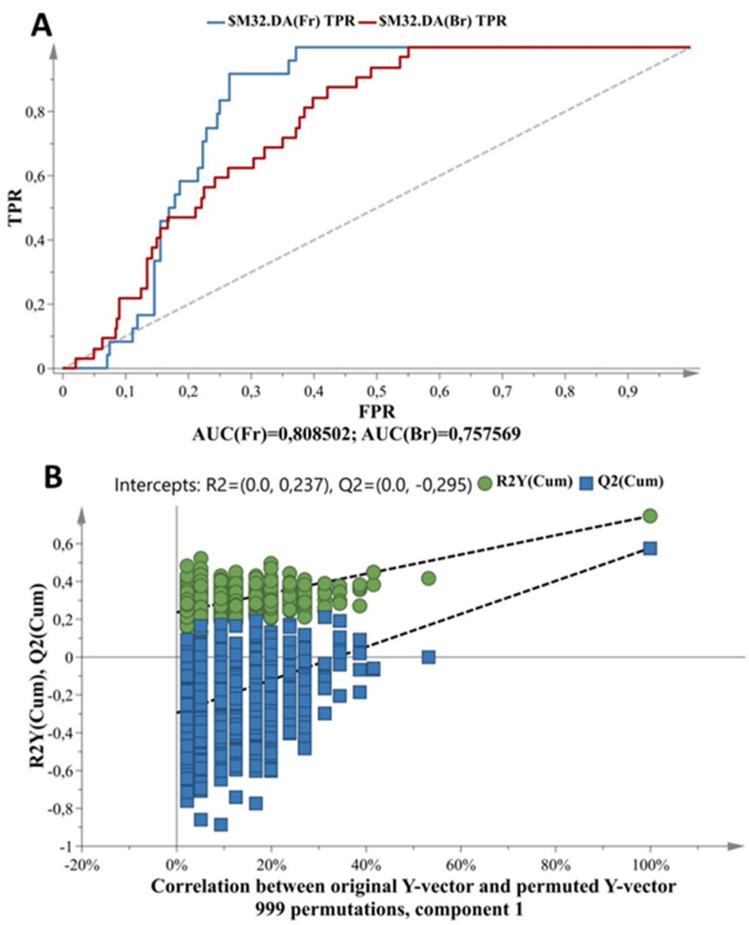

Figure S10. (a) ROC curve AUC 2nd month (Formula-fed) = 0.808 and AUC (Breast Fed) =0.757; **(b)** permutation testing for the OPLS-DA model for formula-fed 3rd day vs 15th day.

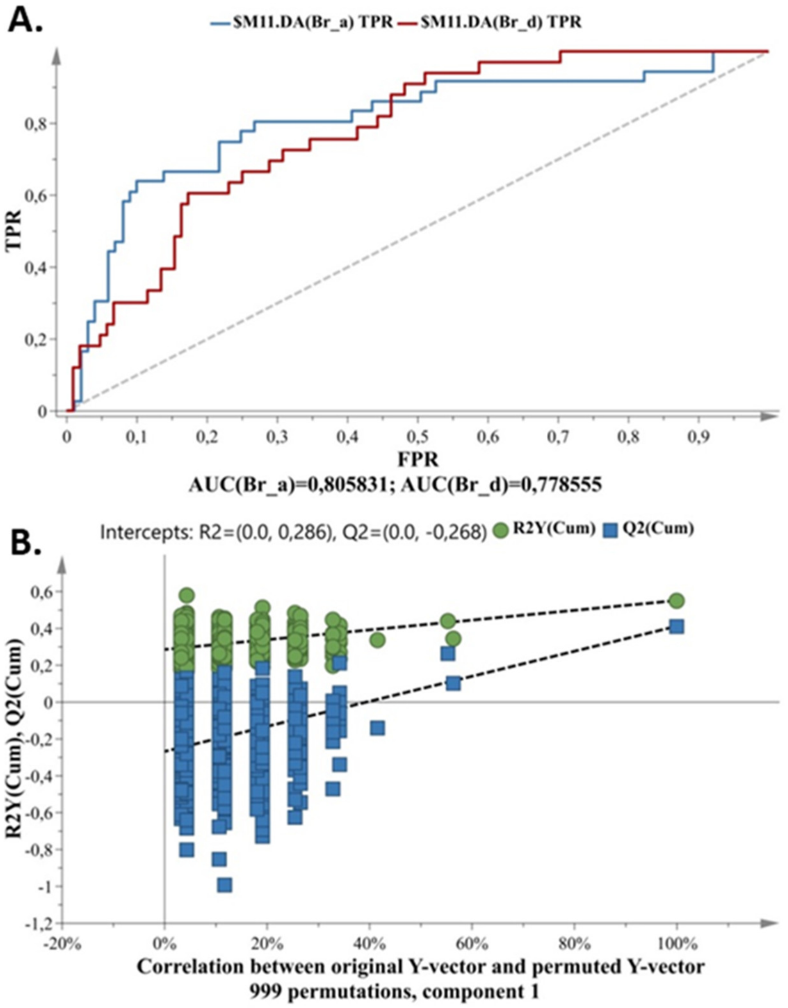

Figure S11. (a) ROC curve AUC (Breastfeeding day 3)=0.805 and AUC (Breastfeeding 3rd month)=0.778, **(b)** permutation testing for the OPLS-DA model in Figure 3.

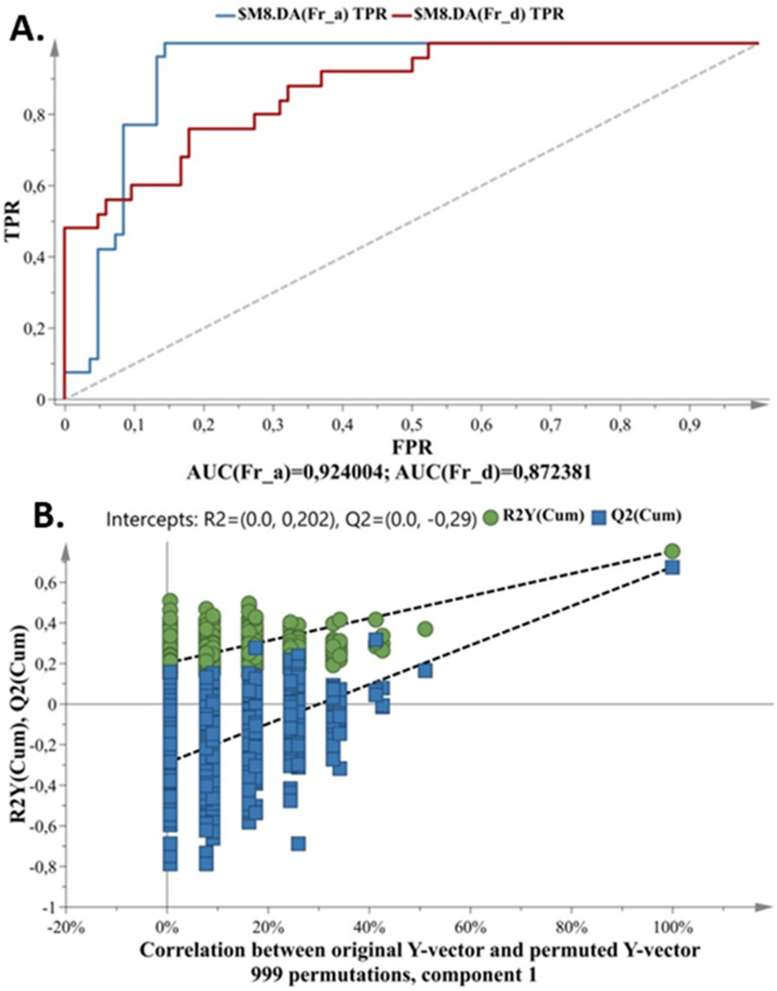

Figure S12. (a) ROC curve AUC (Synbiotic formula day 3)= 0.924 and AUC (Synbiotic formula 3rd month)=0.872, **(b)** permutation testing for the OPLS-DA model in Figure 4.

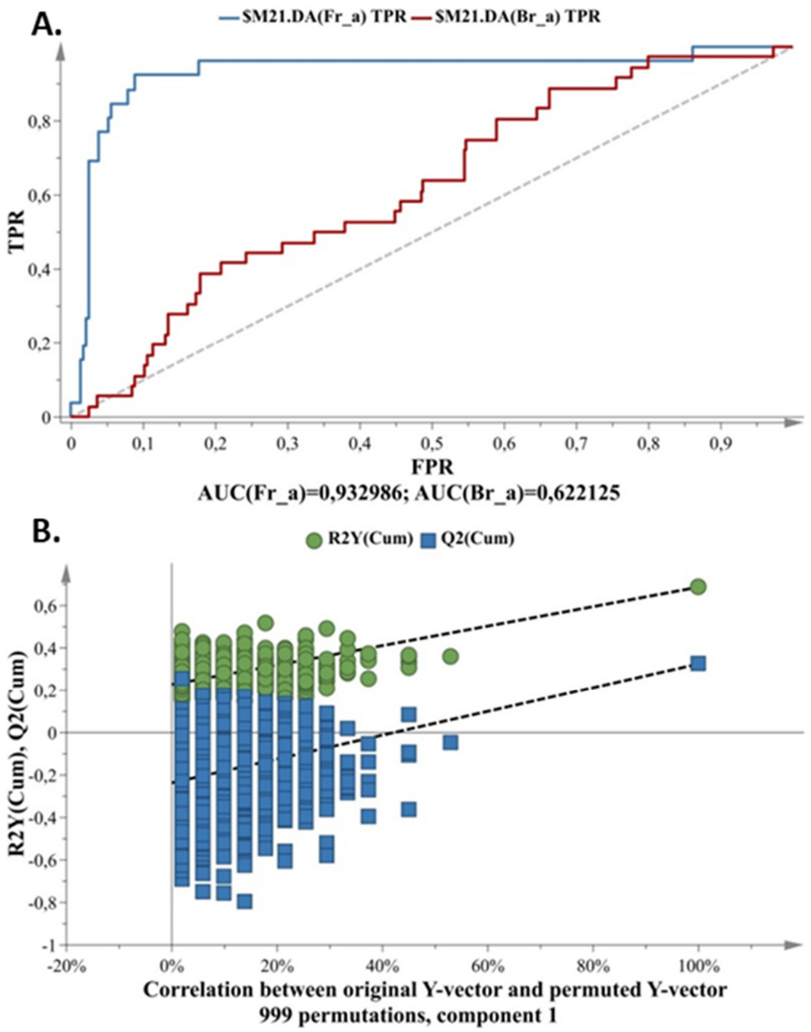

Figure S13. (a) ROC curve AUC (Synbiotic formula day 3)= 0.932 and AUC (Breastfeeding day 3)=0.622, **(b)** permutation testing for the OPLS-DA model of breastfeeding vs formula at 3rd day.

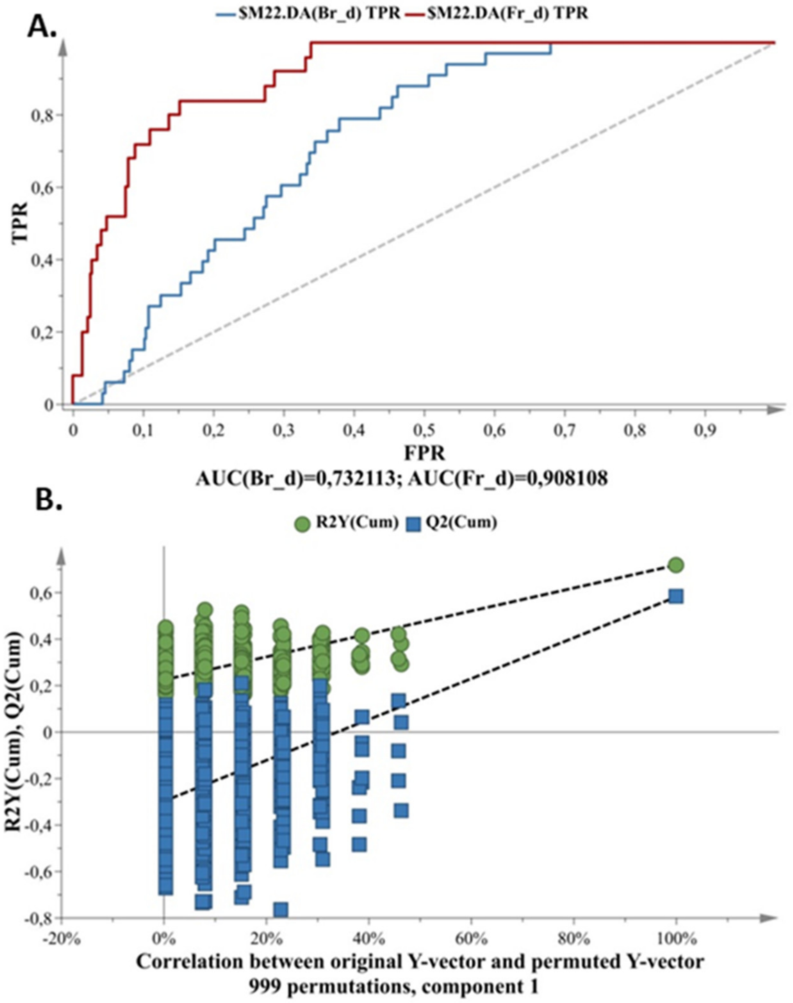

Figure S14. (a) ROC curve AUC (Breastfeeding 3rd month)= 0.732 and AUC (Synbiotic formula 3rd month)=0.908, **(b)** permutation testing for the OPLS-DA model of breastfeeding 3rd month vs synbiotic formula 3rd month.

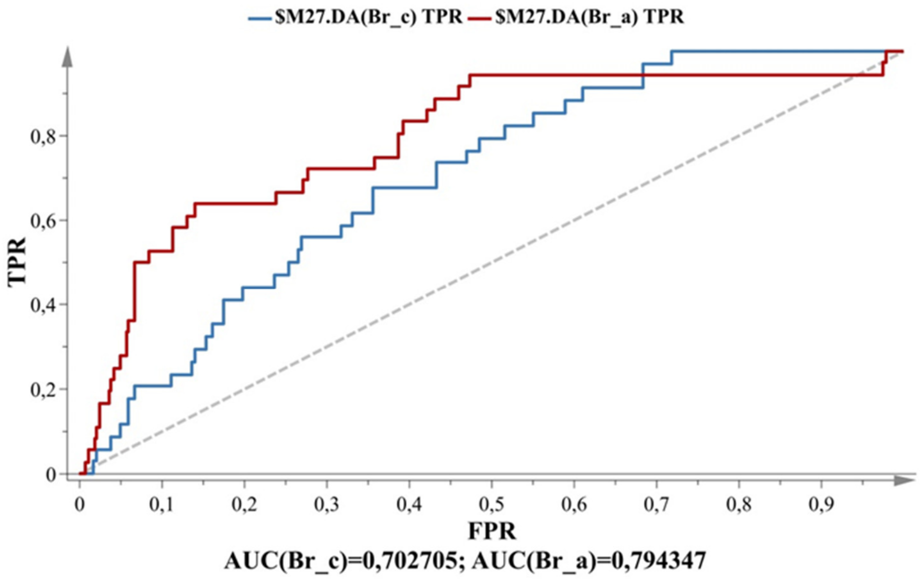

Figure S15. ROC curve AUC (Breastfeeding month 2) = 0.702 and AUC (Breastfeeding day 3) =0.794.

Table S1. Validation metrics for Statistical models of Breast-fed: Br vs Formula-fed: Fr at each time point of the study.

	**PCA**	**PLS[1]**	**OPLS[1]**	**OPLS-DA (SIMCA[2])**
1st Sampling – Day 3<br>Br= 36 vs Fr= 26<br>N=62	R2 = 0.798	R^2 = 0.633<br>Q^2 = 0.344<br>(3 predictors)	R2 = 0.433,<br>Q2 = 0.244<br>N=62	R2Y (cum)=0.69,<br>Q2(cum)=0.33<br>N=51
2nd Sampling – Day 15<br>Br= 34 vs Fr= 31<br>N=65	R2 = 0.858	R2 = 0.573<br>Q2 = 0.395<br>(3 predictors)	R2 = 0.406,<br>Q2 =0.276<br>N=65	R2Y (cum)=0.59,<br>Q2(cum)=0.42<br>N=57
3rd Sampling – Month 2<br>Br= 34 vs Fr= 28<br>N=62	R2 = 0.680	**R2 = 0.834**<br>**Q2 = 0.669**<br>(4 predictors)	R2 = 0.69,<br>Q2 =0.343<br>N=62	**R2Y(cum)=0.75,**<br>**Q2(cum)=0.58**<br>N=56
4th Sampling - Month 3<br>Br= 34 vs Fr= 25<br>N=59	R2 = 0.676	**R2 = 0.922**<br>**Q2 = 0.781**<br>(8 predictors)	R2 = 0.574,<br>Q2 =0. 476<br>N=59	**R2Y(cum)=0.72,**<br>**Q2(cum)=0.58**<br>N=54

[1] p -value = 0.001 for all R^2 and Q^2 of the PLS and OPLS models. [2] Supervised SIMCA models were generated omitting outliers from the PCA models.

Table S2. Validation metrics for statistical models of Breast-fed: Br samples between different time points.

	PCA	PLS	OPLS	OPLS-DA (SIMCA[1])
Day 3 (n= 36) vs Day 15 (n= 34)	R2 = 0.843	R2 = 0.245<br>p-value = 0.05<br>Q2 = 0.018<br>p-value = 0.281(2 predictors)	R2 = 0.283<br>p-value = 0.173<br>Q2 = 0.04<br>p-value = 0.067	OPLS: R2 =0.298, Q2 = 0.0338
Day 3 (n= 36) vs Month 2 (n= 34)	R2 = 0.813	R2 = 0.367<br>p-value = 0.003<br>Q2 = 0.267<br>p-value = 0.001<br>(2 predictors)	R2 = 0.367<br>p-value = 0.003<br>Q2 = 0.25<br>p-value = 0.001	R2X(cum)=0.61,<br>R2Y(cum)=0.48,<br>Q2(cum)=0.33<br>N=64
Day 3 (n= 36) vs Month 3 (n= 34)	R2 = 0.791	R2 = 0.391<br>p-value = 0.001<br>Q2 = 0.264<br>p-value = 0.001<br>(2 predictors)	R2 = 0.391<br>p-value = 0.001<br>Q2 = 0.201<br>p-value = 0.001	R2X(cum)=0.57,<br>R2Y(cum)=0.55,<br>Q2(cum)=0.41<br>N=54
Day 15 days (n=34) vs Month 2 (n= 34)	R2 = 0.858	R2 = 0.227<br>p-value = 0.05<br>Q2 = 0.104<br>p-value = 0.01<br>(2 predictors)	R2 = 0.227<br>p-value = 0.05<br>Q2 = 0.113<br>p-value = 0.003	OPLS: R2 = 0.362, Q2 = 0.183
Day 15 days (n=34) vs Month 3 (n= 34)	R2 = 0.838	R2 = 0.351<br>p-value = 0.002<br>Q2 = 0.187<br>p-value = 0.001<br>(2 predictors)	R2 = 0.267<br>p-value = 0.001<br>Q2 = 0.147<br>p-value = 0.001	OPLS: R2 = 0.489, Q2 = 0.34
Month 2 (n=34) vs Month 3 (n= 34)	R2 = 0.721	R2 = 0.335<br>p-value = 0.504<br>Q2 = 0.106<br>p-value = 0.104<br>(2 predictors)	R2 = 0.313<br>p-value = 0.07<br>Q2 = -0.201<br>p-value = 0.652	-

[1] Supervised SIMCA models were generated omitting outliers from the PCA models.

Table S3. Validation metrics for statistical models of formula-fed: Fr samples between different time points.

	PCA	PLS	OPLS	OPLS-DA (SIMCA[1])
Day 3 (n= 26) vs Day 15 (n= 31)	R2 = 0.68	R2 = 0.516<br>p-value = 0.008<br>Q2 = 0.118<br>p-value = 0.046<br>(3 predictors)	R2 = 0.401<br>p-value = 0.007<br>Q2 = 0.246<br>p-value = 0.002	OPLS: R2 = 0.401,<br>Q2 = 0.228
Day 3 (n= 26) vs Month 2 (n= 28)	R2 = 0.613	R2 = 0.732<br>p-value = 0.001<br>Q2 = 0.416<br>p-value = 0.001<br>(2 predictors)	R2 = 0.732<br>p-value = 0.001<br>Q2 = 0.591<br>p-value = 0.001	R2X(cum)=0.53,<br>R2Y(cum)=0.75,<br>Q2(cum)=0.55<br>n=53
Day 3 (n= 26) vs Month 3 (n= 25)	R2 = 0.644	R2 = 0.816<br>p-value = 0.001<br>Q2 = 0.681<br>p-value = 0.001<br>(4 predictors)	R2 = 0.698<br>p-value = 0.001<br>Q2 = 0.639<br>p-value = 0.001	R2X(cum)=0.51,<br>R2Y(cum)=0.75,<br>Q2(cum)=0.67<br>n=48
Day 15 (n= 31) vs Month 2 (n= 28)	R2 = 0.691	R2 = 0.36<br>p-value = 0.004<br>Q2 = 0.208<br>p-value = 0.002<br>(2 predictors)	R2 = 0.36<br>p-value = 0.003<br>Q2 =0.158<br>p-value = 0.003	OPLS: R2 =0.377,<br>Q2 = 0.215
Day 15 (n= 31) vs Month 3 (n= 25)	R2 = 0.716	R2 = 0.518<br>p-value = 0.002<br>Q2 = 0.382<br>p-value = 0.001<br>(3 predictors)	R2 = 0.433<br>p-value = 0.001<br>Q2 = 0.405<br>p-value = 0.001	PCA: R2 = 0.805<br>OPLS: R2 = 0.492,<br>Q2 = 0.386
Month 2 (n= 28) vs Month 3 (n= 25)	R2 = 0.674	R2 = 0.164<br>p-value = 0.466<br>Q2 = 0.033<br>p-value = 0.091<br>(2 predictors)	R2 = 0.162<br>p-value = 0.462<br>Q2 = 0.024<br>p-value = 0.122	PCA: R2 = 0.768<br>OPLS: R2 = 0.263,<br>Q2 = 0.106

[1] Supervised SIMCA models were generated omitting outliers from the PCA models.

Table S4. Average nutritional composition per 100 mL of human breast milk and formula (Rontamil® Complete 1) [1,2].

	**Formula***	**Breast[1, 2]**
Energy (kcal)	68	63
Protein (g)	1.4	1.04
Carbohydrates (g)	7.8	7.84
Fibre (g)	0.34	1.2-1.4
Fat (g)	3.4	3.0
Saturated Fatty acids (g)	1.0	1.1
MUFA (g)	1.71	1.4
PUFA (g)	0.66	0.5
Linoleic acid (g)	0.52	0.4
α-linolenic acid (mg)	54	17.8
Arachidonic acid -AA (mg)	15.4	15.2
Docosahexaenoic acid – DHA (mg)	15.4	7.6
Minerals		
calcium (mg)	47	20-25
phosphorus (mg)	29	12-14
magnesium (mg)	5	3.0-3.5
iron (mg)	0.5	0.03-0.07
zinc (μg)	0.5	0.1-0.3
manganese (μg)	7.9	0.3-0.4
copper (μg)	50	0.01-0.03
iodine (μg)	12.6	14-15
sodium	23	15-25
potassium	74	40-55
chloride	54	40-45
selenium (μg) 2.71.0-2.5fluoride6.8-Other		

total nucleotides (mg)	2.2	
Non-protein nitrogen (g)		0.18
Probiotics (Bifidobacterium animalis) (cfu/g)	1.00E+07	

*100mL formula milk is prepared by mixing 13.5 g powder with 90 ml water

Table S5. Data comparison between formula-fed and breast-fed groups.

	**Formula-fed**	**Breast-fed**
Birth weight Mean (g)	3,287	3,298
Birth weight Std Dev (g)	410	339
Sex Ratio (F/M)	0.78	0.59
Delivery Mode Ratio (Physical/Cesarean)	0.88	1.69

References

1. Koletzko, B. Human Milk Lipids. *Ann Nutr Metab* **2016**, *69 Suppl 2*, 28–40, doi:10.1159/000452819.
2. Kim, S.Y.; Yi, D.Y. Components of Human Breast Milk: From Macronutrient to Microbiome and MicroRNA. *Clin Exp Pediatr* **2020**, *63*, 301–309, doi:10.3345/cep.2020.00059.
